# Discussion

**Published:** 1918-11

**Authors:** 


**Discussion.**

Major General Sir John Rose Bradford, R. A. M. C. In the autumn of 1914 there was a considerable number of cases of a remarkable form of bronchitis, which we were not familiar with in civil life. It was a malady which was very fatal; it ran a very prolonged course of 3 to 6 weeks, with a very high temperature of typhoid-like character, and characterized by expectoration of a very large quantity of green sputum.

This disease did not exist in 1915. It occurred again in 1916. The medical officers were more familiar with it then than they were in 1914, when they had diagnosed it as pneumonia, typhoid, etc. It did not occur to any appreciable extent in 1917.

It was not a streptococcus infection, but an infection in which the bacillus of influenza was the most common organism, though it occurred at times when influenza was not prevalent. Only a few cases occurred this spring and summer when influenza was prevalent.

Another clinical phenomenon was the very large number of cases of dry pleurisy which we have seen, particularly in the first two years of the war, — I mean pleurisy not in association with tuberculosis. These cases were far more prevalent than the ordinary forms of pleurisy.

As regards pneumonia, we have had very little in the British Army. I have seen comparatively little pneumonia as regards the percentage of men. Some colonials, particularly Australians, suffer very much from it.

However, there are many points of interest as regards pneumonia. Very few cases in the British Army have been running a typical course. In the early days of the war the men had a very fine physique, and it was expected that pneumonia would run the familiar course, but it was very common to see some cases running a long course and others with an imperfect resolution. The resolution was sometimes delayed 6 weeks, yet there was no error of diagnosis and no question of empyema. I have been profoundly impressed by the anomalous course of these cases of pneumonia.

Variety of organisms : Certain limited series of observations were made in one of the areas where I had my work. The prevalent pneumococci were the usual type I, type II, and type IV. In a series of over 50 cases there was not a single type III; they were mostly type I or type II.

A certain limited number of cases that Captain Wilson examined were dependent on streptococcus. Those cases showed a remarkable sputum of citron color, but I do not believe that citron-colored sputum is a particularity of streptococcus infections.

I should think that a considerable amount of work might be done with regard to the bacteriology of pneumonia, and that there is room for investigation as regards the clinical side of pneumonia, because there has been a surprisingly large proportion of cases that have run a course with which one is quite unfamiliar in civil life. There is room for investigation both on the bacteriological and the clinical side.

The mortality of cases where there was complication of measles or other infectious diseases has not been high.

Major Joslin, M. C. Last winter, being in charge of the medical service of a base hospital in America, I had the same problems to meet that were presented in other base hospitals.

We had to deal with a large number of respiratory infections. The cases were all more or less crowded, and we had a certain amount of cross infection. I will not go into the measures of isolation because we practised all those described to-day.

There are various ways of meeting this infection or transmission of organism from one person to another. We know that we can either keep the organisms in the carriers, which is the function of the mask and cubicle, or practise the method of giving more room by having less patients in a ward and the beds further apart.

We have practised preventive inoculation, so that patients are not susceptible, but there are as yet no very satisfactory methods of inoculating patients for the prevention of streptococcus and pneumococcus infections.

We were rather led to try methods for clearing the carriers, and we practised a good deal of this in various ways during last winter. We were somewhat impressed with the results, without being convinced that we had accomplished a good deal.

Our attention was called to the fact that following an epidemic of measles of 4000 cases we had a large proportion of otitis media—several hundred cases at one time. They were pretty serious and we had some deaths following complications.

We thought that it might be of some value to try to keep the naso-pharyngeal region clear. The most effective way was to use a solution of carbolic acid in glycerine and in oil, applied by swabbing out the throat and putting drops in the nose. Almost immediately the epidemic of otitis media decreased.

Up to the middle of the winter we had no severe epidemics of streptococcus infections. After this, we began to have cases of erysipelas and two or three cases of streptococcus meningitis. In the spring, after a large epidemic of influenza, we began to have cases of streptococcus pneumonia.

Our experience was different from that of other camps because streptococcus pneumonia had been more common in other hospitals than with us, and the type of pneumonia had changed. When erysipelas occurred we cultured all patients, doctors, and nurses in the ward. We were all thoroughly and vigorously sprayed and nearly all carriers seemed to be cleared, so that repeated cultures failed to show streptococci on subsequent inoculation.

Then we thought it might be of some use to attempt something with pneumococcus. We did not start as early as we might and for certain reasons were unfortunate. But we went over the various regiments in camp, culturing them for the presence of pneumococcus. We divided the men into squads of 16, because we had just put up some small shacks containing 16 men comfortably. Each squad was given a different type of treatment, and cultured every third day for twenty or thirty days to be thoroughly sure how we were getting along in regard to pneumococcus. We found hat our solution of carbolic acid was fairly effective. We kept on the men for some time and found only one persistent and permanent carrier; all the others cleared up pretty rapidly.

An important feature was that our epidemic of pneumonia subsided very rapidly, and whether these measures were of any avail I don't know; but if we had taken measures of this kind at the beginning of the winter, possibly the severity of our epidemic might have been to some extent controlled.

Medecin-Major Cattel. It has fallen to my lot to make a large number of autopsies, and to be in possession of the records of autopsies as made in France at the present time. The chief complications of pneumonia have been broncho-pneumonia and croupous pneumonia, a proportion running into empyema. During studies of these cases of empyema, the following facts were prominently brought out: —

When the surgeon or physician chose a place for tapping, he almost uniformly counted the spaces and the ribs wrong and went down lower than he intended. It is the practice to tap lower on the left side than on the right. If the physician decided to use the eighth rib he almost uniformly opened the 9th rib or the 9th interspace, in making his incision lower than he thought. There have been a large number of cases where injuries have been done to the abdominal viscera, where the liver has been punctured, and where peritonitis has occurred.

One of the particularities of the epidemics of empyema in the States was that there was a closure of the pocket very early in the disease. This small pocket, often containing organisms, is not opened by the best surgical means; it is at an inaccessible point and difficult to reach.

If you draw the same picture over here in France, you merely have to change the word " measles " and introduce the word " influenza ", but with the difference that the number of cases of croupous pneumonia is larger following influenza than broncho-pneumonia. You may expect to find more fatal cases of croupous pneumonia than of streptococcus pneumonia.

You are most likely familiar with Professor Mac Callum's paper showing that it is possible from a macroscopic study of a section of lung to tell the presence of hemolytic streptococcus. With experience, in 95 o/o of the cases one can tell whether one is dealing with streptococcus, pneumococcus, or a a mixed type.

In regard to where this epidemic is arising all that it is wise to say at the present time is that it is arising, at one of the base ports, and that persons are there being placed on cars already infected, are taken off the cars at various stations in France, and are being treated at various hospitals on the way.

In one series of pneumonia, in which the influenza bacillus has been found in almost 80 o/o of the cases, type III makes a peculiar form of lesion that cannot be definitely recognized.

Therefore, from the autopsies it appears that the influenza bacillus, and that a secondary means of infection is the chief agent, streptococcus haemolyticus.

Colonel Thayer, M. C. In dealing with pneumonia associated with influenza and other types of pneumonia, it is not the same story repeated everywhere? My impression is that this croupous pneumonia — or post-influenza pneumonia — is very likely that which was seen in the epidemic of 1889.

One point has not been brought up, but it should be mentioned, in regard to the empyema which so often follows pneumonia. We are all familiar with the method of treatment, and I hope there are not many men in the A. E. F. who are going to tap too low and penetrate the abdominal cavity. A man who cannot do this correctly should go home.

We are all familiar with the post-pneumonic empyema, which is benign if operated early, and which shows a very thick greenish pus. We are most of us familiar with the streptococcus empyema, which in ordinary practice is not very common, and which produces there a heavy sputum, very high fever, and a rapid accumulation of fluid only faintly cloudy. Most of us have been in the habit of feeling that such cases should be opened at the earliest possible moment and that every day lost is a grave danger to the patient. One point that is very important is that it has been unanimously concluded that it is unwise to operate surgically at once on streptococcus empyema, and that one should always aspirate several times. After several days, when the fluid has become thick, it is wise to operate. Cases which are drained immediately do not do well, but die. Cases which are aspirated several times before operation, show a very fair proportion of recoveries.

This brought back to my mind the only instance of streptococcus empyema which I have had in my private practice. The patient had been in the hands of half a dozen doctors, and when I saw him I felt he was going to die. He was operated on immediately and he recovered, possibly because the disease was not quickly recognized and he was not operated on earlier. I think evidence shows it to be unwise to drain surgically a streptococcus empyema; it is better to aspirate several times and operate later.

In regard to the question of medical technique, this is an old story; we physicians are deplorably lax in our ward technic and throw up our hands when suggestions are made that we should do certain things; we say that they are impossible, that they would complicate things, etc. What would we have said 40 years ago if one had mentioned the precautions that surgeons take every day in the operating room? Who knows what we may consider necessary 30 years hence in an operating room? The proper screening, proper masking, and care of patients when admitted to hospitals are precautions that are desirable, and we have good evidence that these things help. We may be very severely criticized in the future if we do not make every effort to carry them out. We too often wander in the wards in our ordinary everyday clothes, examine typhoid fever patients, and put our hands in beds, only washing our hands after seeing half a dozen patients. We are often utterly careless in the most simple precautions. We should always wash our hands, wear gowns, and be very particular about everything in regard to respiratory infections.

Lieutenant-Colonel Gerald B. Webb, M. C. I regret that Colonel Thayer did not speak to you concerning the point about which I whispered to him, and which has been missed in this excellent discussion, as I know how much better Colonel Thayer could have placed it before you. I refer to the simple matter of abundance of fresh air and ventilation.

I have just returned from a brief visit to England where I saw large numbers of cases of pneumonia dying in base hospitals, these cases having developed on board our transports. The types appeared to be both broncho-pneumonia and croupous pneumonia from the post mortems that I observed. The diseases were very swift in their progress, and there had been no time for the development of empyemata. Multiple lobes were usually involved. It was observed that on ships on which the transport surgeons had refused to accept soldiers with evidences of catarrhal colds little disease developed. From one transport where such care in the boarding of troops was possibly not insisted upon, very large numbers of serious chest cases developed, with a high percentage of deaths, and other conditions may have existed on individual transports inducing fatigue.

When strolling through Westminster Abbey I noticed on the walls a memorial to sir John Pringle. Pringle, in the 17th century, observed that in the wars the English were fighting on the Continent, when men with medical and surgical conditions were placed in buildings where doors and windows had been destroyed, almost invariably good recoveries were made; whereas when perfect buildings were used, serious results were seen. Pringle became famous on the question of abundance of fresh air and ventilation, and even described principles for the proper ventilation of troop ships. It is interesting for us to remember that he toured on the Continent with Benjamin Franklin, and I do not doubt they were congenial spirits.

Observing so many men in hospital wards coughing violently without even protecting the mouth, it has occurred to me that a setting-up exercise, consisting in making men hold the hand in front of the mouth and cough into the hand, would be excellent hygienic instruction for our troops. I should like to see this suggestion issued as a general order to the men in the line.

There is no doubt, in regard to the movement of troops, especially across the Atlantic, that the greatest care should be observed in the separation of men with symptoms of catarrhal conditions before accepting them on board the transports. There is also no doubt that in the crowding in transports and barracks we should insist on far better ventilation than is now being carried out. After the present epidemic, it will be important that slowly convalescent chest conditions — broncho-pneumonia and pleurisies — are not confused with pulmonary tuberculosis. Persistent negative sputa and careful observation for several weeks will obviate such mistakes.

Major Seale Harris, M. C. The principal question that has been brought out in the discussion is that of prevention. One aspect of prevention has not been discussed, and that is the value of education. We ought to start an educational campaign that would reach every man in the A. E. F. Keep the men from getting infection, go after carriers that you know about, and consider that every man is under suspicion of being a carrier of streptococcus haemolyticus and influenza bacillus until proved otherwise. Every man ought to be taught how to take care of his sputum and prevent others from being infected.

This is as much part of the line officers' duty as it is that of the medical officers. Medical officers should first be taught and impressed with the necessity of preventing these respiratory infections. Conferences should be held with the line officers, in which it should be explained that they have an enemy within their lines which will cause as many deaths as the enemy in the trenches. These line officers should teach the soldiers how to take care of sputum. The disposal of sputum is a very important thing. If all sputum from streptococcus carriers were destroyed, that would stop infection; and if every man were taught that when he coughs, even if he is well himself, he is liable to give his neighbor a disease, or streptococcus infection, he would be more careful about coughing and spitting.

Major Joslin said that all the medical officers in his hospital were found to be streptococcus carriers. In this room there has been quite a lot of coughing, and I have noticed that very few men have taken the precaution of preventing droplets from getting on everyone in the room; a few have put their handkerchiefs to their mouths, but more did not.

In the army it is a question of discipline. Company officers should say to the men " If you expectorate on the ground or on the floor, you will be disciplined ". The men should expectorate in antiseptic fluids. Men with coughs should be given paper cups which could be destroyed after use. We need a campaign of education, like the one in regard to tuberculosis, which would teach these men that all who expectorate may give diseases to other men, even though they are perfectly well themselves.

Major HAVEN EMERSON. The carrier problem is in its infancy from a bacteriological point of view. It has not been proved that carriers play an important part in outdoor, open-air conditions, however much they do in wards.

The first demonstration of the cubicle system, as an adequate method of preventing acute respiratory diseases, was made at the Pasteur Institute in Paris where it has been operated for ten years. It is used in various hospitals in America, and is essential for the care of diphtheria, measles, and scarlet fever. As Colonel Thayer said, we have only just begun to conceive medical aseptic technic. It has been mentioned that if sputum were blue there would be a tinge of blue over most appurtenances of hospital wards. Although you may interfere with infection by partitions between the beds, the only effective prevention is decent ward technic by doctors and nurses.

If you insist about the washing of hands and avoiding of contact of nurses and doctors, you can gradually reduce the height of the partition between the beds, then you can try a bar between the beds, then a red string will be found to do just as well, if the technic of personal cleanliness is carried out adequately.

It is well to recognize streptococcus infections as a text to talk from, but that is not what we are suffering from now. I would try to impress upon you that we are at the threshold of an epidemic of influenza similar to that which occurred from 1889 to 1892. It is not imported solely from the United States in ships, it began here. On April 15th we had our first epidemic south of Bordeaux, and since then it has gone through the entire A. E. F. Reports of this disease have come not only from Spain, but from the Italian front, from Cuba, and from Sweden. An important document has just reached us from Switzerland, and records there indicate that between 80 and 90 o/o of the entire mobilized Swiss Army were infected in one month after having escorted French and American prisoners from Germany. The rate of mortality of pneumonia complicating it has been 40 o/o, and the epidemic was so important that for a period the Franco-Swiss frontier was closed as a sanitary measure. It is worth noting that Switzerland has published an official report of the disease with details of treatment, because they have recognized that they are the means of spreading this epidemic. It has affected London very seriously, the B. E. F. have had it, and it spread subsequently to the A. E. F. in the south of France. Two or three months before we began to get cases back from the U. S., steamers were held in quarantine in New York before the passengers were allowed to land.

It is a mistake to think the streptococcus problem is in the U. S.; it is here, and we are faced with a most serious problem from the point of view of sickness and death rate.

It is important for us to realize that it is not going to be settled in hospital wards. It is a field problem, a problem of sanitation, of getting support for individual resistance by proper care of individual soldiers by squad leaders, by platoon leaders and by surgeons of regiments who must know their men as old artillery and cavalry men knew their horses.

At present it is obvious that many men are being sacrificed to pneumonia because of the poor judgment used in places where they are being transported. Transportation should be avoided if possible. There is no disease for which transportation is so serious. We have records here showing how the incidence of influenza is accompanied by coincidence in the rate of pneumonia, and by an increased death rate front pneumonia.

One other respiratory disease has shown an increase, and that is meningitis. The British E. F. and A. E. F. have an average of 8 to 12 cases of pneumococcus meningitis. The week before last they had 51 cases, and for the first 5 and 6 days of last week they had 60 cases. It is merely the inevitable increase of pneumococcus infections on people whose upper respiratory tract is inflamed in the course of infection which prevails everywhere. We must prevent contact.

Sanitary reports show that out of 30 epidemics in one region, 27 are influenza, and 3 ordinary infectious diseases. There are communities of 2000 people in various parts where this same pneumonia has occurred with 20 deaths in a week; this has occurred very seriously among new recruits of the French Army. The same has occurred in the French military and civili n population who have no contact with American troops. Therefore it is not only being brought over in ships.

It is very important to realize that we are in the midst of a pandemic like the one of 1889; it spreads with greater rapidity now because of the difference in transportation. It is extremely rapid in transmission, and we are now having about 4000 to 4500 cases a week, which are sufficiently severe to go into hospital, with a death rate of 30 o/o from complications. I would urge you to take up all measures which have been found practical. Tired, ill-fed and over-crowded soldiers, and those driven with work, are more susceptible to the disease than men who are adequately rested. Wherever you can improve the resistance of the individual you are doing more than could be accomplished by attempting to cut off infection.

Major Carlson. Of course the value of fresh air is recognized, but you must remember that the soldiers on transports coming to England are placed in a position where they cannot get fresh air; they are badly fed and sometimes get seasick. They are brought over in all kinds of conditions which would not be used in peace times. Under those conditions we are going to have more epidemics. I wonder whether it would not be possible to apply the isolating methods on the transports that are being applied in hospitals (masking and possibly spraying). I understand that the British authorities have had some success with spraying. The conditions on transports are perfectly unsanitary; sometimes the troops cannot get up on deck more than 2 or 3 hours a day, and carriers will infect the whole mass, particularly when severe weather comes, as there is no facility for warming the ships.

Major Leconte. I should like to say a word on the side of the Navy in this discussion. A good deal has been said about transports, and Major Cattell spoke of one port as the infecting source of troops. I would like to give you a picture of that port. It is one of the finest deep water ports in Europe, and certainly the best in France. Accommodation is extremely limited and few ships can dock there, these being only the smaller freight ships. Facilities for landing troops are also extremely limited, so limited that they have to work night and day. Its camping facilities are likewise extremely limited, and at times when large transports are landing troops by thousands the railroad is inadequate, and we have thousands of men living in camps where tents have been pitched under rainstorms and in the mud. During the last 5 weeks the transports have been loaded as full as they could be. The life of the men on board is led almost wholly in their sleeping quarters. Sleeping accommodation is so limited that the men have to take turns for getting in the bunks. Ventilation is practically nil. The submarine zone has extended for considerable distance, and during the night every porthole is closed and every particle of light is shut off from the exterior, which means no ventilation where the men sleep. Many of the men sleep on the deck, even on unsheltered decks, in order that they may get air. In one transport recently there were hundreds of men sleeping on deck; they appointed a guard with an officer to walk among these sleeping men at night so that those who rolled out of their blankets could be covered again. Three doctors attended to this duty and all of them died. In one transport there were 800 cases of illness, dozens of cases of pneumonia, and in some instances there have been as many as 30 and 40 deaths during the passage.

At the same time, these sick men were landed in already overcrowded hospitals, and many had to be cared for in their quarters.

This particular port has spread the disease throughout France. Troops are being shipped from this camp as rapidly as the railroad can take them, but when a convoy arrives many of the men have to stay for days and days in these surroundings. Hospital accommodation is limited and the medical force in charge is overworked.

I think that the cases that have originated in this port have been brought from America; certainly those that have proved fatal have been brought from America. I have known regiments to leave a camp in a perfect state of health, to take troop trains in America and arrive on board their steamer after 12 or 24 hours. The first day out a few cases of influenza developed, the second day out there were scores of cases, and the third day hundreds of cases. With these cases of influenza comes virulent pneumonia. Whether a regiment that leaves its camp well has acquired disease in the troop trains is a question which is very serious, and I am told that troop trains are so limited in number that they are acting like a shuttle from camp to steamer. In the dust of such conveyances streptococcus and other organisms have been discovered.

One word about the epidemic of influenza with streptococcus pneumonia which came in the 1920 recruits of the French Navy, breaking out in one of the barracks. There was a large number of cases of influenza with streptococcus pneumonia, and the mortality was 30 to 40 o/o from streptococcus pneumonia. Four hundred deaths occurred. The outbreak was stopped by taking the recruits, dividing them in groups of 50 to 100 men and sending them into barracks out of town. In these barracks the medical officers took the temperature and pulse of the well troops night and morning. When any individual showed a temperature, or an increase of pulse without temperature, he was separated from the rest. The disease was soon stamped out because the medical officers hunted for it. The civil population did not have streptococcus pneumonia; they had hundreds and thousands of cases of ordinary grippe but very few cases, or deaths, from pneumonia. The American troops quartered in this same base did not have the outbreak; they were not contaminated by the French troops; and at that time we had very few cases of pneumonia among our troops or sailors. The fleet has not yet had its attack; up to the present time it has had a few cases of grippe but no pneumonia.

On one transport the mortality among those who developed pneumonia was between 60 and 90 o/o. I think that the keynote has been struck by Colonel Webb when he says that the infection starts in America and not here.

